# Evaluation of Industry Payments to US Advanced Practice Clinicians in 2021

**DOI:** 10.1001/jamanetworkopen.2022.42869

**Published:** 2022-11-18

**Authors:** Armaan Singh, Max J. Hyman, Parth K. Modi

**Affiliations:** 1 Pritzker School of Medicine, The University of Chicago, Chicago, Illinois; 2 The Center for Health and the Social Sciences, The University of Chicago, Chicago, Illinois; 3 Section of Urology, Department of Surgery, The University of Chicago, Chicago, Illinois

## Abstract

**Importance:**

Advanced practice clinicians (APCs) are a growing part of the US health care system, and their financial relationships with pharmaceutical and medical device companies have not been well studied.

**Objectives:**

To examine the value, frequency, and types of payments made to APCs and the association of state scope-of-practice laws with these payments.

**Design, Setting, and Participants:**

This cross-sectional study used 2021 Open Payments Program data to analyze payments from pharmaceutical or medical device companies to physicians or APCs between January 1 and December 31, 2021. Doctors of medicine and osteopathy were categorized as physicians, and nurse practitioners, physician assistants, clinical nurse specialists, certified registered nurse anesthetists, certified nurse midwives, and anesthesiologist assistants as APCs.

**Main Outcomes and Measures:**

The total value and total number of payments were calculated in aggregate and per clinician for each type of APC, all APCs, and physicians. These calculations were repeated by submitting manufacturer, form of payment, nature of payment, and state scope-of-practice law for nurse practitioners, physician assistants, and physicians.

**Results:**

A total of 412 000 physicians and 232 000 APCs collectively received $1.99 billion in payments from industry in 2021, of which APCs received $121 million (6.1%). The median total value of payments per clinician for physicians was $167 (IQR, $45-$712) and for APCs was $117 (IQR, $33-$357). The median total number of payments per clinician was equal for physicians and APCs (n = 4). The most common payments to APCs included food and beverage ($69 million [57.6%]), compensation for services other than consulting ($32 million [26.4%]), and consulting fees ($8 million [6.6%]). Advanced practice clinicians in states with the most restrictive scope-of-practice laws received 15.9% lower total value of payments than those in the least restrictive states (*P* = .002). Physician assistants received 7.6% (*P* = .005) higher value and 18.1% (*P* < .001) greater number of payments than nurse practitioners.

**Conclusions and Relevance:**

In this cross-sectional study, 232 000 APCs collectively received $121 million in industry payments in 2021. The frequency of industry interactions with APCs was similar to that for physicians, but the average value was lower. The greater value of payments to APCs who practice in states with the least restrictive scope-of-practice laws suggests that industry payments may be related to clinician autonomy.

## Key Points

**Question:**

How much, how many, and what types of payments did advanced practice clinicians (APCs) receive from pharmaceutical and medical device companies in 2021?

**Findings:**

In this cross-sectional study of industry payments to physicians and APCs in the US, 232 000 APCs collectively received $121 million in nonresearch payments from industry in 2021; over the same period, 411 739 physicians received $1.8 billion. The average number of payments per individual was similar for APCs and physicians, and nurse practitioners and physician assistants in states with the least restrictive scope-of-practice laws were paid more than those in the most restrictive states.

**Meaning:**

The findings of this study suggest that more than one-third of APCs in the US received payments from pharmaceutical and medical device companies in 2021. The financial relationships between APCs and industry are important to examine as the role of APCs in the US health care system grows.

## Teaser

This cross-sectional study examines the number and amount of payments made by pharmaceutical and medical device companies to advance practice clinicians (APCs).

## Introduction

Industry payments to physicians have become a common marketing strategy among pharmaceutical and medical device companies, totaling more than $3.6 billion in 2019.^1,2^ These payments are distinct from those for research and may include consulting and speaker fees, gifts, or meals. Studies have shown an association between industry payments and physicians’ clinical research, prescribing patterns, and medical device selection.^3,4,5^ In response to concerns about physician integrity and transparency from patients and policy makers,^6,7^ the US Congress passed the Physician Payments Sunshine Act in 2010. This act created the Open Payments program and required the public reporting of every payment greater than $10 from a pharmaceutical or medical device company to a physician.^8^

The Open Payments program has improved our understanding of the extent and effects of industry payments to physicians, but much less is known about industry payments to advanced practice clinicians (APCs). Advanced practice clinicians function in both primary and specialty care settings and provide care that often overlaps with that provided by physicians, including acute care, prescribing medications, and chronic disease management.^9^ Therefore, it is possible that payments will influence APC behavior in the same way as they influence physician behavior. The importance of studying industry payments to APCs reflects their growing role in the US medical workforce. Advanced practice clinicians made up 29.1% of the primary care workforce in 2010,^10^ and the numbers are expected to grow annually through 2030 for nurse practitioners (NPs) (6.8%) and physician assistants (PAs) (4.3%). This estimate projects that 68% of clinicians entering the workforce from 2016 to 2030 will be APCs.^9^ Therefore, as the number of states allowing unsupervised clinical practice for APCs continues to increase, there is a growing need to understand their financial relationships with industry.^11^

The Open Payments Program collected data on industry payments to APCs for the first time in 2021. This change in data collection was due to the passage of the 2018 Support Act bill (P.L. 115-271) by the US Congress,^12^ which was enacted in response to growing concern around the opioid epidemic. The bill eliminated APCs’ exemption from data collection as part of an effort to increase transparency regarding marketing to all clinicians. Herein, we provide what we believe to be the first analysis of payments to APCs using these national data. We examined the value, frequency, and types of payments made to APCs, as well as the association between state scope-of-practice laws and the value and frequency of the payments they receive. We hypothesized that APCs with more independence (ie, working in states with less restrictive scope-of-practice laws) will receive more payments from industry than their colleagues in more restrictive states.

## Methods

The University of Chicago Institutional Review Board exempted this study from full review and the requirement for informed consent because the data are publicly available. This study followed the Strengthening the Reporting of Observational Studies in Epidemiology (STROBE) reporting guideline.^13^

### Study Population

This study used the 2021 Open Payments program data published by the Centers for Medicare & Medicaid Services.^8^ The data include every submitted payment from an applicable manufacturer or group-purchasing organization to a physician, teaching hospital, or APC between January 1 and December 31, 2021. Payment data are organized into 3 data sets: (1) general payments not in connection with a research agreement or protocol, (2) research payments in connection with a research agreement or protocol, and (3) physician ownership or investment interest information about physicians or their immediate family members who hold an ownership or investment interest in an applicable manufacturer or group-purchasing organization. This study used the general payments data set. We did not include research payments in this study because APCs received less than 0.01% of research payments in 2021.^2^

We included any payment labeled as a new, undisputed, and nondelayed submission and received by a physician or APC in the US or its territories with a valid National Provider Identifier number. The physicians category of the provider type variable includes doctors of medicine (MD) and osteopathic medicine (DO), dentists, podiatrists, optometrists, and chiropractors. The APC category includes NPs, PAs, clinical nurse specialists, certified registered nurse anesthetists, certified nurse midwives, and certified anesthesiologist assistants. We categorized payments to MDs and DOs as physician payments and payments to any type of APC as APC payments.

### Payment Data

Each observation in the data set consists of a single payment or consolidation of payments from a submitting manufacturer or group-purchasing organization to a clinician. Each observation includes the payment amount in US dollars, the form of the payment (eg, cash, stock), the nature of the payment (eg, food and beverage, consulting fee) (eTable 1 in the Supplement), and the number of payments. The states in which each clinician is licensed to practice is also listed for each payment.

### Outcomes of Interest

The primary outcomes include the total amount of payments, total number of payments, median total value of payments per clinician, and median total number of payments per clinician. These outcomes were calculated for each type of APC, all APCs, and physicians. We also examined submitting manufacturer, product category, form of payment, and nature of payment for NPs, PAs, and physicians.

For NPs, we classified state scope of practice as level 1 (full scope of practice with no physician supervision or collaborative practice agreement with a physician required), level 2 (collaborative practice agreement with a physician required to diagnose or manage illnesses), and level 3 (direct physician supervision or involvement required to diagnose and treat illnesses).^11^ Twenty-six states were designated as level 1, 13 states as level 2, and 11 states as level 3. For PAs, we classified state scope of practice as level 1 (collaborative arrangement), level 2 (supervisory arrangement without physician cosignature required on medical records, and level 3 (supervisory arrangement with physician cosignature required on a certain percentage or number of medical records).^11^ For PAs, 5 states were designated as level 1, 27 states as level 2, and 18 states as level 3 (eTable 2 in the Supplement).

As a secondary outcome, we calculated the proportion of each APC type, all APCs, and physicians who were practicing in 2021 and received an industry payment. The numerators in these calculations were the number of unique clinicians paid as defined by the number of unique National Provider Identifier in our data. The denominators—the number of unique clinicians practicing—required we access another data set: the National Plan & Provider Enumeration System file from the Centers for Medicare & Medicaid Services.^14^ This file contains every enumerated clinician (ie, National Provider Identifier), and we selected those (1) within the US or its territories, (2) that were neither deactivated before January 1, 2021, nor activated after December 31, 2021, and (3) had a physician or APC taxonomy code. The National Plan & Provider Enumeration System file also lists the reported sex (male, female) of each clinician.^15^ As an additional secondary outcome, we examined the differences in payments to physicians and APCs by sex.

### Statistical Analysis

The total value of payments and total number of payments to APCs are examples of count data (ie, nonnegative integers) with a positive skew. Therefore, the associations between state scope-of-practice laws and total value or number of payments to APCs were examined with negative binomial regression. Each observation in the regression was an APC and the model included the clinician type (NP vs PA), category of state scope of practice, and state. Standard errors were clustered by state to account for correlation within groups. Testing was 2-sided and statistical significance was set at α = .05. Data cleaning and analysis were performed in R, version 3.6.3 (R Foundation for Statistical Computing), Stata, version 17.0 (StataCorp LLC), and SAS, version 9.4 (SAS Institute Inc).

## Results

### Overall Payments

A total of 643 820 unique clinicians collectively received $1.99 billion in payments from pharmaceutical and medical device companies in 2021 (Table 1). We found that approximately 36.7% of physicians and 37.5% of APCs received at least 1 payment from industry. Physicians received $1.87 billion (93.9%) and APCs received $121 million (6.1%), of which NPs received $79 million (65.3%), PAs received $39 million (32.2%), certified registered nurse anesthetists received $2 million (1.7%), clinical nurse specialists received $1 million (0.8%), and certified nurse midwives and certified anesthesiologist assistants received less than $1 million. The median total value of payments per physician was $167 (IQR, $45-$712), and the median total value of payments per APC was $117 (IQR, $33-$357). The median total number of payments per physician and per APC was 4 (physician IQR, 1-17; APC IQR, 1-14).

**Table 1.** Industry Payments in 2021

Outcomes	Physician	APC	NP	PA	CRNA	CNS	CNM	AA
No. of unique clinicians paid	411 739	232 081	150 010	67 888	12 176	999	939	69
No. of unique clinicians practicing	1 120 957	618 145	369 734	164 348	63 346	7873	9723	3121
Proportion of unique clinicians paid, %	36.7	37.5	40.6	41.3	19.2	12.7	9.7	2.2
Total value of payments, $	1 834 545 747	120 752 315	78 849 740	38 921 419	1 575 449	1 166 933	221 884	16 891
Total No. of payments	7 433 278	3 606 117	2 338 980	1 192 453	39 592	25 800	9084	208
Total value of payments per clinician, $								
Median (IQR)	167 (45-710)	117 (33-357)	122 (35-363)	133 (38-446)	30 (17-63)	48 (19-121)	29 (17-69)	27 (20-46)
Mean (SD)	4458 (201 184)	517 (3454)	524 (3660)	584 (3195)	101 (1479)	377 (6046)	77 (410)	82 (308)
Total No. of payments per clinician								
Median (IQR)	4 (1-17)	4 (1-14)	4 (1-14)	4 (1-17)	1 (1-2)	1 (1-3)	1 (1-2)	1 (1-1)
Mean (SD)	18 (36)	15 (31)	16 (30)	18 (34)	2 (4)	3 (6)	2 (2)	1 (1)

Abbreviations: AA, anesthesiologist assistant; APC, advanced practice clinician; CNM, certified nurse midwife; CNS, clinical nurse specialist; CRNA, certified registered nurse anesthetist; NP, nurse practitioner; PA, physician assistant.

### Form, Nature, Product Category, and Manufacturers of Payments

The forms (ie, types) of payments to APCs included cash or cash equivalent and in-kind items and services. The form of payments to physicians also included stock, stock option, any other ownership interest, and dividend, profit, or other return on investment. The median payments for cash or cash equivalent payments to physicians were $98 (IQR, $28-$781); to NPs, $47 (IQR, $20-$124); and to PAs, $51 (IQR, $21-$130). The median payments for in-kind items and services payments to physicians were $142 (IQR, $41-$489); to NPs, $119 (IQR, $34-$336); and to PAs, $125 (IQR, $36-$411) (Table 2).

**Table 2.** Form of Industry Payments in 2021^a^

Form of payment	No. (%)		Median (IQR)	
	Total value of payments, $	Total No. of payments	Total value of payment per clinician, $	Total No. of payments per clinician
Nurse practitioner				
Cash or cash equivalent	33 201 187 (42)	191 457 (8)	47 (20-124)	2 (1-4)
In-kind items and services	45 648 553 (58)	2 147 523 (92)	119 (34-336)	4 (1-14)
Physician assistant				
Cash or cash equivalent	14 376 401 (37)	94 760 (8)	51 (21-130)	2 (1-4)
In-kind items and services	24 545 018 (63)	1 097 693 (92)	125 (36-411)	4 (1-16)
Physician				
Cash or cash equivalent	1 567 245 137 (85)	1 029 064 (14)	98 (28-781)	2 (1-6)
In-kind items and services	225 587 000 (12)	6 403 547 (86)	142 (41-489)	4 (1-16)
Stock	8 450 177 (<0.5)	130 (<0.5)	50 117 (12 125-182 895)	1 (1-2)
Stock option	9 710 902 (1)	213 (<0.5)	9600 (2500-106 518)	1 (1-2)
Any other ownership interest	17 631 819 (1)	45 (<0.5)	314 381 (134 500-810 545)	1 (1-1)
Profit, dividend, or other ROI	5 920 712 (<0.5)	279 (<0.5)	7718 (3000-67 713)	3 (2-4)

Abbreviation: ROI, return on investment.

^a^ Values in parentheses are column percentages, and they may not sum to 100 due to rounding.

The 3 highest value natures (ie, sources) of payments to physicians included royalties ($480 million [26.9%]), consulting fees ($385 million [21.6%]), and compensation for services other than consulting ($368 million [20.6%]). The 3 highest value natures of payments to NPs included food and beverage ($45 million [57.7%]), compensation for services other than consulting ($23 million [28.9%]), and consulting fees ($5 million [6.4%]). For PAs, these payments included food and beverage ($23 million [60.0%]), compensation for services other than consulting ($9 million [22.5%]), and consulting fees ($3 million [6.6%]). Food and beverage accounted for 97% of the total number of payments to NPs and PAs and 91% to physicians (Table 3). Overall, payments to APCs were most commonly for food and beverage ($69 million [58.6%]), compensation for services other than consulting ($32 million [26.8%]), and consulting fees ($8 million [6.4%]).

**Table 3.** Nature of Industry Payments by Clinician Type in 2021^a^

Nature of payment	Total value of payments, $			Total No. of payments		
	NP	PA	Physician	NP	PA	Physician
Acquisitions	0	15 011 (<0.5)	250 930 749 (14)	0	1 (<0.5)	651 (<0.5)
Charitable contribution	12 475 (<0.5)	0	196 281 (<0.5)	19 (<0.5)	0	27 (<0.5)
Non-CME speaker fees	22 802 134 (29)	8 759 009 (23)	367 780 276 (20)	21 281 (1)	8076 (1)	194 025 (3)
CME speaker fees	219 652 (<0.5)	144 132 (<0.5)	9 575 338 (1)	328 (<0.5)	277 (<0.5)	6179 (<0.5)
Consulting fees	5 011 090 (6)	2 580 695 (7)	385 676 670 (21)	4852 (<0.5)	2666 (<0.5)	152 437 (2)
Ownership or investment	0	0	17 548 316 (1)	0	0	518 (<0.5)
Debt forgiveness	3793 (<0.5)	23 426 (<0.5)	1 717 668 (<0.5)	54 (<0.5)	34 (<0.5)	634 (<0.5)
Education	1 254 051 (2)	1 579 522 (4)	53 770 119 (3)	20 522 (1)	10 757 (1)	107 468 (1)
Entertainment	5930 (<0.5)	961 (<0.5)	62 014 (<0.5)	96 (<0.5)	13 (<0.5)	607 (<0.5)
Food and beverage	45 482 153 (58)	23 365 559 (60)	157 927 141 (9)	2 274 572 (97)	1 160 561 (97)	6 728 333 (91)
Gift	16 885 (<0.5)	4977 (<0.5)	304 122 (<0.5)	813 (<0.5)	331 (<0.5)	5867 (<0.5)
Grant	16 817 (<0.5)	104 225 (<0.5)	21 731 314 (1)	17 (<0.5)	9 (<0.5)	2192 (<0.5)
Honoraria	809 352 (1)	455 585 (1)	33 653 436 (2)	1116 (<0.5)	446 (<0.5)	17 535 (<0.5)
Supply or device loan	109 000 (<0.5)	21 317 (<0.5)	2 252 056 (<0.5)	642 (<0.5)	523 (<0.5)	3081 (<0.5)
Royalty or License	0	12 423 (<0.5)	480 292 031 (26)	0	4 (<0.5)	12 471 (<0.5)
Travel and Lodging	3 106 408 (4)	1 854 578 (5)	511,28 214 (3)	14 668 (1)	8755 (1)	201 253 (3)

Abbreviations: CME, continuing medical education; NP, nurse practitioner; PA, physician assistant.

^a^ Values in parentheses are column percentages, and they may not sum to 100 due to rounding. Definitions and examples of each nature of payment may be found in eTable 1 in the Supplement.

We found that most of the total value and number of payments to NPs and PAs were for pharmaceutical products (Table 4). For physicians, however, medical devices were responsible for 56% of the total value of payments. The manufacturers responsible for the greatest number of payments, total value of payments, and number of unique clinicians paid are detailed in eTable 3 in the Supplement. Although male physicians received a higher median number (5 [IQR, 2-19]) and value ($201 [IQR, $53-$898]) of industry payments than female physicians (3 [IQR, 1-13] and $125 [IQR, $35-$433]), the opposite was true for APCs (eTable 4 in the Supplement). Female APCs received a median of 4 (IQR, 1-14) payments with median value of $120 (IQR, $34-$361), and male APCs received a median of 3 (IQR, 1-12) payments with a median total value of $99 (IQR, $29-$323).

**Table 4.** Industry Payments by Product Category in 2021^a^

Product category	Total value of payments, $^b^			Total No. of payments^b^		
	NP	PA	Physician	NP	PA	Physician
Biologic	10 749 429 (14)	7 339 086 (21)	148 032 157 (11)	283 439 (12)	218 401 (19)	1 031 521 (15)
Medical device	7 192 787 (10)	5 703 931 (16)	740 629 565 (56)	159 661 (7)	109 999 (10)	1 338 508 (19)
Drug	50 321 745 (68)	19 199 904 (54)	368 608 795 (28)	1 728 765 (76)	769 745 (67)	4 390 993 (62)
Medical supply	165 328 (<0.5)	52 519 (<0.5)	1 942 740 (<0.5)	3483 (<0.5)	1703 (<0.5)	12 473 (<0.5)
Multiple^c^	949 567 (1)	571 581 (2)	5 982 437 (<0.5)	57 428 (3)	31 832 (3)	187 784 (3)
Unspecified^d^	5 073 011 (7)	2 742 578 (8)	58 325 722 (4)	37 708 (2)	19 266 (2)	129 474 (2)

Abbreviations: NP, nurse practitioner; PA, physician assistant.

^a^ Nonproduct payments were not included in this analysis (4.1% of payments).

^b^ Values in parenthesis are column percentages, and they may not sum to 100 due to rounding.

^c^ Multiple product category represents consolidated payments with 2 or more product categories listed.

^d^ Unspecified product category represents payments with no product category listed.

### Scope of Practice

The total value of payments was 6.3% higher for PAs compared with NPs (incidence rate ratio [IRR], 1.063; 95% CI, 1.006-1.123; *P* = .03) (Table 5). States with the most restrictive scope-of-practice laws (level 3) were associated with 16.3% lower total value of payments to APCs compared with the states with the least restrictive laws (IRR, 0.837; 95% CI, 0.750-0.934; *P* = .001). Physician assistants received 15.6% more payments compared with NPs (IRR, 1.156; 95% CI, 1.103-1.213; *P* < .001). There was no association between state scope-of-practice laws and the total number of payments to APCs. In an exploratory post hoc analysis of NPs in the least vs most restrictive scope-of-practice states, speaker’s fees represented a higher proportion of the total value of payments (35% vs 26%) and food and beverage a smaller proportion (49% vs 62%).

**Table 5.** Industry Payments to Nurse Practitioners and Physician Assistants Are Associated With State Scope of Practice^a^

Variable	IRR (95% CI)	*P* value
**Total value of payments**		
Clinician type		
NP	1 [Reference]	NA
PA	1.063 (1.006-1.123)	.03
Scope of practice		
Level 1	1 [Reference]	NA
Level 2	0.982 (0.886-1.098)	.73
Level 3	0.837 (0.750-0.934)	.001
**Total No. of payments**		
Clinician type		
NP	1 [Reference]	NA
PA	1.156 (1.103-1.213)	<.001
Scope of practice		
Level 1	1 [Reference]	NA
Level 2	0.969 (0.912-1.031)	.32
Level 3	0.939 (0.878-1.004)	.06

Abbreviations: IRR, incidence risk ratio; NA, not applicable; NP, nurse practitioner; PA, physician assistant.

^a^ Scope of practice equal to level 1 is least restrictive and equal to level 3 is most restrictive. Regressions include state fixed effects and SEs clustered by state.

## Discussion

We examined industry payments to APCs in the 2021 Open Payments Program and arrived at 3 major findings. First, APCs collectively received $121 million in industry payments in 2021, which was 6.1% of the total value of payments to all clinicians in 2021. A similar proportion of APCs and physicians had interactions with industry, and the median number of payments per clinician was identical for physicians and APCs. Second, payments to APCs were most commonly for food and beverage ($69 million [58.6%]), compensation for services other than consulting ($32 million [26.8%]), and consulting fees ($8 million [6.4%]). Third, APCs in the most restrictive scope-of-practice states received a lower value of payments compared with those in the least restrictive states.

Previous studies examining industry payments to physicians have found associations between payments and physician prescribing patterns,^4^ formulary recommendations,^16^ clinical research,^17^ or clinical practice guidelines.^18^ Although US national data for payments to nonphysicians did not exist before the 2021 Open Payments data, there has been evidence in the US and internationally that nurses have interactions with industry representatives that are similar to physician-industry interactions.^19,20^ Furthermore, a study of APCs from Washington, DC, reported that NPs and PAs who received gifts from the pharmaceutical industry had higher cost prescription drug claims.^3^ In addition, qualitative research suggests that APCs have positive sentiments toward companies marketing to clinicians despite their recognition of bias-related issues.^21^ Therefore, industry payments to APCs may be associated with APC behavior that mirrors that with physician behavior, and since APCs make up a growing proportion of the medical workforce, their financial relationships with industry are increasingly relevant to patient care and health policy.

A consequence of the Open Payments program and disclosure of industry payments to physicians has been the growth in scholarship and awareness of industry financial interactions with clinicians. As APCs were not previously included in these data, the extent to which APCs are aware of the implications of industry relationships and the public reporting of these data is not clear. There are limited publications regarding training in the ethics of industry-clinician financial relationships for APCs; the American Medical Association has supported medical student education on this topic.^22^ It is likely that, with the inclusion of APCs in the Open Payments program reporting requirements, awareness will increase.

The makeup of payments to APCs differed in several ways compared with payments to physicians. Most of the value of payments to APCs was related to food and beverage, consulting fees, or speaker fees. Physicians, however, had a substantial proportion of payments for acquisitions and royalty or licensing fees, which were essentially absent for APCs. Overall, the most frequent interactions with industry were for food and beverage payments for physicians, NPs, and PAs. Similarly, physicians had much higher payments in cash, stock, and ownership interests. For in-kind items and services (including food), the number and value of interactions were similar for all 3 groups. These findings suggest that, although there are more highly paid outliers among physicians (often related to ownership and consulting), the most common types of interactions are very similar for physicians and APCs.

The sources of payments to physicians and APCs differed, likely due to differences in their clinical contexts. We found that most (56%) of the total value of payments to physicians was related to medical devices. This was a much smaller component of the total value of payments to NPs (10%) and PAs (16%). However, this difference was much less for the total number of payments, which were mostly related to drug marketing for all clinicians. Taken together, these data suggest that industry interactions are broadly similar for APCs and physicians, but for a smaller number of high-value interactions with physicians related to medical devices and often for ownership and consulting services.

Our finding that APCs in the least restrictive scope-of-practice states received greater value in marketing payments is consistent with other literature suggesting that industry payments influence prescribing decisions.^4,23^ Directing payments to prescribers more likely to independently make prescription decisions is logical. Nonetheless, these findings are hypothesis generating and additional research is warranted to understand any associations between payments and APC behavior. Furthermore, future research examining the association of industry payments with APC specialties, responsibilities, and professional titles would be useful, as previous studies have shown that physicians in positions of influence often receive greater industry payments.^24^

Gender differences in industry payments to physicians are well established.^25,26,27^ In this study, we also found that male physicians received higher numbers and value of industry payments compared with female physicians in 2021. However, this pattern was reversed for APCs. Although this study could not elucidate reasons for this finding, one possibility is that more female clinicians are in leadership or prominent positions among APCs. Further studies with additional data on the clinical and professional characteristics of APCs are needed to elucidate this finding.

### Limitations

This study has limitations. First, this study was cross-sectional and descriptive. Therefore, it cannot causally explain the reasons for or effects of industry payments to APCs. Second, the practice context of these APCs cannot be inferred from these data. Unlike physicians who are identified with a specialty, APCs do not have well-defined specialty categorizations in the National Plan & Provider Enumeration System or Open Payments Program databases (eg, options for PAs include PA, PA medical, or PA surgical). Furthermore, NP specialties (eg, family or acute care) cannot reliably define their practice contexts. We also must consider the possibility of inaccuracies in the Open Payments data. We excluded disputed and delayed submissions to limit this possibility, but these data aggregate millions of records and may contain coding or administrative errors. In addition, our models assessing the association between state scope-of-practice and APC payments likely exclude confounding variables that may explain our results. The scope-of-practice analysis should be considered as hypothesis generating.

## Conclusions

More than one-third of APCs in the US collectively received $121 million in industry payments in 2021. Payments to APCs were higher in states with the least restrictive scope-of-practice laws and were smaller in value compared with payments to physicians. Our findings present a major step forward in helping stakeholders understand how industry payments may influence the growing APC workforce. For policy makers, our results reveal that APCs have similar levels of exposure to industry marketing efforts as physicians. For clinicians, these results are a reminder that APCs, like physicians, must remain cognizant of the influence their industry relationships may have on their practice patterns.

## Notes

Supplement. **eTable 1.** Definitions and Examples of Natures of Payment **eTable 2.** State Scope-of-Practice Laws **eTable 3.** Top Five Manufacturers in Total Value of Payments, Total Number of Payments, and Number of Unique Clinicians Paid in 2021 **eTable 4.** Industry Payments in 2021 by Clinician Gender
