# Supplementary material for: Evaluation of Industry Payments to US Advanced Practice Clinicians in 2021
Source: JAMA Netw Open. 2022 Nov 18;5(11):e2242869. doi: 10.1001/jamanetworkopen.2022.42869 (PMC9674997; doi:10.1001/jamanetworkopen.2022.42869)
Supplement: Supplement. — eTable 1. Definitions and Examples of Natures of Payment eTable 2. State Scope-of-Practice Laws eTable 3. Top Five Manufacturers in Total Value of Payments, Total Number of Payments, and Number of Unique Clinicians Paid in 2021 eTable 4. Industry Payments in 2021 by Clinician Gender [file jamanetwopen-e2242869-s001.pdf]

Supplementary Online Content

Singh A, Hyman MJ, Modi PK. Evaluation of industry payments to US advanced practice clinicians in 2021. *JAMA Netw Open*. 2022;5(11):e2242869.
doi:10.1001/jamanetworkopen.2022.42869

eTable 1. Definitions and Examples of Natures of Payment

eTable 2. State Scope-of-Practice Laws

eTable 3. Top Five Manufacturers in Total Value of Payments, Total Number of Payments, and Number of Unique Clinicians Paid in 2021

eTable 4. Industry Payments in 2021 by Clinician Gender

This supplementary material has been provided by the authors to give readers additional information about their work.

© 2022 Singh A et al. *JAMA Network Open.*

eTable 1: Definitions and Examples of Natures of Payment

Nature of Payment	Definition	Example
Acquisitions	Buyout payments made to covered recipients who have ownership interest in a company that has been acquired.	A drug manufacturer buys a share of ownership in a company that is at least partially owned by a physician or a physician’s immediate family member.
Charitable contribution	A payment or transfer of value made to an organization with tax-exempt status under the Internal Revenue Code of 1986. Charitable contributions do not include payments or transfers of value that would be more specifically described by one of the other payment categories.	A medical device manufacturer donates funds to a teaching hospital to help pay for a health education program.
Non-CME speaker fees	Includes payments that a company makes to physicians for speaking, training, and education engagements that are not for continuing education.	-
CME speaker fees	Compensation for serving as faculty or as a speaker for medical education program.	Drug company Y gives money to a teaching hospital to help pay for the hospital’s annual course for its physicians. The course is an update on the latest treatments for diseases.
Consulting fee	A payment that a company makes to a physician for advice and expertise about a medical product or treatment. Consulting fees are typically arranged with a written agreement between a company and physician based on the company’s particular business needs. These payments often vary depending on the consulting physician’s expertise.	Company A has developed a drug to treat patients with a particular disease and wants advice from physicians on how to design a large study to test the drug on patients. Dr. J has a large number of patients with this disease and has experience researching medicines that could treat this condition. Company A asks Dr. J if she would spend about 10 hours per month to work with other physicians to create a new research study. Dr. J agrees and is paid for her time.
Ownership or investment	Ownership or investment interest currently held by physicians and teaching hospitals, as well as ownership or investment interest that could potentially be held by physicians and teaching hospitals.	Dr. M hears about a new antibiotic that Pharmaceutical Company F is developing. Dr. M thinks the drug might become successful and asks if he can invest in Company F. Company F agrees, and Dr. M ends up owning a percentage of Company F.
Debt forgiveness	Forgiving the debt of a covered recipient, a physician owner, or the immediate family of the physician.	A physician owes Company A an amount of money for medical supplies. Company A forgives the debt so that the physician can keep the supplies without providing payment.

© 2022 Singh A et al. *JAMA Network Open.*

Education	Payments or transfers of value for classes, activities, programs, or events that involve learning or teaching a profession skill. This payment can include things like textbooks and medical journal articles.	Companies that produce or sell drugs or devices for a particular medical condition may offer textbooks to physicians, free of charge, related to the latest treatments for that condition.
Entertainment	Attendance at recreational, cultural, sporting or other events that would generally have a cost.	A physician receives tickets to a local football game from a device manufacturer that owns season tickets.
Food and beverage	Food and beverage.	A drug manufacturer salesperson asks to speak with a physician about a new drug. The salesperson and physician meet for lunch, and the salesperson pays for the meal.
Gift	A general category which includes anything a company provides to a physician or teaching hospital that does not fit into another category.	Promotional items such as clocks or flash drives that have the company's name printed on them.
Grant	A payment to a physician or teaching hospital to support a specific cause or activity.	Company G is a medical device manufacturer. Company G gives a grant to a teaching hospital to pay for special training for physicians who want to learn more about how to perform surgeries to give patients Company G's device.
Honoraria	Similar to consulting fees, but generally reserved for a brief, one-time activity. Another distinction is that honoraria are generally provided for services without a set price.	A medical device manufacturer representative goes to a medical meeting. At the meeting, the representative asks physicians for an hour of their time to talk about features they would like to see on a particular medical device. The representative pays each physician a one-time honorarium.
Supply or device loan	The loan of supplies or a device for 91 days or longer.	A device manufacturer lends one of its devices to a teaching hospital for 120 days.
Research	Payments for different types of research activities, including the time a physician spends enrolling patients in studies for new drugs or devices. Research payments can include direct compensation to physicians, funding for research study coordination and implementation, or payments to study participants to cover expenses associated with the study.	A physician wants to study treatments for a specific ailment. Pharmaceutical Company H is interested in the results and offers to provide funds for the incentives the physician uses to recruit participants.
Royalty or license	Payments based on sales of products that use a physician's intellectual property.	A device manufacturer may promise a certain amount of payment in royalties – 1% of all device sales, for example – to a physician who worked with the device manufacturer to invent a new product.

© 2022 Singh A et al. *JAMA Network Open.*

Space rental or facility fees	Payments for fees associated with renting a space or facility (such as a teaching hospital).	A drug manufacturer wants to offer training to physicians on how to administer a drug. The drug manufacturer pays a teaching hospital to reserve space within the hospital to conduct the training.
Travel and lodging	Any compensation for costs associated with travel, such as hotel fees, airfare, mileage, and cab fare.	A medical device company offers yearly training events for physicians on how to use their device on patients. The medical device company pays for the physicians' airfare and hotel rooms when the physicians travel to the medical device company's headquarters for the training.

Source: https://www.cms.gov/OpenPayments/Natures-of-Payment. Accessed 9/24/22.

© 2022 Singh A et al. *JAMA Network Open.*

eTable 2: State Scope-of-Practice Laws

**State**	**NP**	**PA**
Alabama	2	3
Alaska	1	1
Arizona	1	2
Arkansas	2	2
California	3	3
Colorado	1	3
Connecticut	1	2
Delaware	1	2
Florida	3	2
Georgia	3	2
Hawaii	1	3
Idaho	1	2
Illinois	2	1
Indiana	2	2
Iowa	1	3
Kansas	2	3
Kentucky	1	3
Louisiana	2	3
Maine	1	2
Maryland	1	2
Massachusetts	1	2
Michigan	3	1
Minnesota	1	2
Mississippi	2	2
Missouri	3	3
Montana	1	3
Nebraska	1	3
Nevada	1	3
New Hampshire	1	2
New Jersey	1	3
New Mexico	1	1
New York	2	2
North Carolina	3	2
North Dakota	1	2
Ohio	2	3
Oklahoma	3	2
Oregon	1	2
Pennsylvania	2	3
Rhode Island	1	2
South Carolina	3	3
South Dakota	2	2
Tennessee	3	3
Texas	3	2
Utah	1	2
Vermont	1	3
Virginia	3	2
Washington	1	2
West Virginia	2	1
Wisconsin	2	2
Wyoming	1	2

© 2022 Singh A et al. *JAMA Network Open.*

Notes: Scope-of-practice equal to (Level) 1 is least restrictive and equal to (Level) 3 is most restrictive.
Abbreviations: NP, Nurse practitioner; PA, Physician Assistant; NA, Not Applicable.

© 2022 Singh A et al. *JAMA Network Open.*

eTable 3: Top Five Manufacturers in Total Value of Payments, Total Number of Payments, and Number of Unique Clinicians Paid in 2021

Total Value of Payments					
NP		PA		Physician	
**Manufacturer**	**$ (%)**	**Manufacturer**	**$ (%)**	**Manufacturer**	**$ (%)**
AbbVie	10,095,949 (13)	AbbVie	4,707,625 (12)	Medtronic	130,564,570 (7)
Allergan	4,833,698 (6)	Allergan	2,914,283 (7)	Hill-Rom	110,558,487 (6)
AstraZeneca Pharmaceuticals	3,939,842 (5)	Janssen Pharmaceuticals	1,884,813 (5)	Stryker Corporation	102,758,825 (6)
Novo Nordisk	3,711,045 (5)	Galderma Laboratories	1,350,625 (3)	AbbVie	70,461,970 (4)
Janssen Pharmaceuticals	3,569,239 (5)	Novo Nordisk	1,327,948 (3)	DePuy Synthes	56,806,167 (3)
**Total Number of Payments**					
**NP**		**PA**		**Physician**	
**Manufacturer**	**N (%)**	**Manufacturer**	**N (%)**	**Manufacturer**	**N (%)**
AbbVie	338,580 (14)	AbbVie	171,194 (14)	AbbVie	802,529 (11)
Novo Nordisk	130,071 (6)	Janssen Pharmaceuticals	66,711 (6)	AstraZeneca Pharmaceuticals	378,845 (5)
AstraZeneca Pharmaceuticals	128,455 (5)	Novo Nordisk	55,663 (5)	Janssen Pharmaceuticals	359,870 (5)
Janssen Pharmaceuticals	114,434 (5)	AstraZeneca Pharmaceuticals	52,878 (4)	Novo Nordisk	285,047 (4)
Eli Lilly and Company	86,106 (4)	Eli Lilly and Company	45,537 (4)	Amgen	236,520 (3)
**Number of Unique Clinicians Paid**					
**NP**		**PA**		**Physician**	
**Manufacturer**	**N (%)**	**Manufacturer**	**N (%)**	**Manufacturer**	**N (%)**
AbbVie	45,192 (30)	AbbVie	19,839 (29)	AbbVie	97,608 (24)
AstraZeneca Pharmaceuticals	28,929 (19)	Janssen Pharmaceuticals	14,336 (21)	Janssen Pharmaceuticals	74,647 (18)
Janssen Pharmaceuticals	28,584 (19)	Pfizer	11,474 (17)	Pfizer	67,140 (16)
Novo Nordisk	24,726 (16)	Eli Lilly and Company	11,036 (16)	AstraZeneca Pharmaceuticals	64,612 (16)

© 2022 Singh A et al. *JAMA Network Open.*

Eli Lilly and Company	22,844 (15)	Amgen	10,950 (16)	Merck Sharp & Dohme	54,918 (13)

Notes: Values in parenthesis are column percentages..
Abbreviations: NP, Nurse practitioner; PA, Physician assistant.

© 2022 Singh A et al. *JAMA Network Open.*

eTable 4: Industry Payments in 2021 by Clinician Gender

Outcomes	Physician			APC		
	**Male**	**Female**	**Unknown**	**Male**	**Female**	**Unknown**
Number of Unique Clinicians Paid	280,747	130,347	1399	41,170	189,283	334
Number of Unique Clinicians Practicing	704,266	416,691	NA	116,592	501,553	NA
Proportion of Unique Clinicians Paid	39.9%	31.3%	NA	35.3%	37.7%	NA
Total Value of Payments	$1,631,589,127	$197,409,873	$5,546,747	$23,094,088	$97,597,040	$61,187
Total Number of Payments	5,589,385	1,832,745	11,148	644,524	2,959,413	2,180
Average Value of Payments	$292	$108	$498	$36	$33	$28
Total Value of Payments per Clinician						
25%ile	$53	$35	$20	$29	$34	$20
Median	$201	$125	$52	$99	$120	$45
75%ile	$898	$433	$172	$323	$361	$130
Mean (SD)	$5,813 (243,443)	$1,525 (12,261)	$3,965 (62,767)	$541 (3725)	$508 (3383)	$183 (653)
Total Number of Payments per Clinician						
Q1	2	1	1	1	1	1
Median	5	3	2	3	4	2
Q3	19	13	5	12	14	5
Mean (SD)	20 (40)	14 (30)	8 (19)	15 (32)	15 (30)	7 (14)

Abbreviations: APC, Advanced practice clinician; SD, Standard deviation; NA, Not Applicable.

© 2022 Singh A et al. *JAMA Network Open.*
